# Supplementary material for: Quantification of All-Trans Retinoic Acid by Liquid Chromatography–Tandem Mass Spectrometry and Association with Lipid Profile in Patients with Type 2 Diabetes
Source: Metabolites. 2021 Jan 19;11(1):60. doi: 10.3390/metabo11010060 (PMC7835841; doi:10.3390/metabo11010060)
Supplement: Supplementary file 1 [file metabolites-11-00060-s001.pdf]

Supplementary Table S1. Assessment of *at*RA stability upon UV-exposure. Stability is defined as the mean of the quantified concentration of three spiked concentrations (nominal) in human artificial plasma samples exposed to UV-light (254 nm) for the given period of time (n=3).

**Nominal concentration [pg mL^{-1}]**	**UV-exposure at 254 nm [min]**	**Measured concentration [pg mL^{-1}]**
600	60	560.8 ± 57.3
600	120	490.6 ± 78.1
600	180	341.4 ± 76.0
600	360	205.5 ± 39.7

Supplementary Table S2. Mean baseline characteristics and *at*RA levels of the control group (as shown in Table 4) and patients with well-adjusted / poorly controlled type 2 diabetes. All parameters were determined prior to collection. Data are mean ± SD; § $p = 0.053$, *$p < 0.05$, **$p < 0.01$, ***$p < 0.001$ vs. controls. All other parameters were statistically not significant ($p > 0.05$).

	**Controls (n=20)**	**Type 2 Diabetes (n=24)**	**Type 2 Diabetes (n=15)**
Sex [% male]	63.8	65.3	66.6
Age [years]	42.1 ± 12.3	49.6 ± 15.5	60.5 ± 6.13 ***
BMI [kg m^{-2}]	24 ± 2.5	31.3 ± 6.1 ***	24.9 ± 1.8
Blood glucose [mg dL^{-1}]	97.1 ± 12.3	186.3 ± 84.3 ***	136.3 ± 48.2 ***
HbA1c [%]	5.7 ± 0.8	11.2 ± 1.6 ***	6.7 ± 1.5 *
CRP [mg L^{-1}]	1.2 ± 1.1	12.9 ± 8.6 ***	1.3 ± 1.7
Total Cholesterol [mg dL^{-1}]	168.4 ± 19.9	256.1 ± 74.5 ***	179.5 ± 37.3
Triglycerides [mg dL^{-1}]	137.7 ± 24.2	285.3 ± 86.4 ***	118.3 ± 44.9
LDL [mg dL^{-1}]	82.7 ± 25.7	167.5 ± 73.4 ***	101.1 ± 30.6
HDL [mg dL^{-1}]	58.2 ± 15.9	31.5 ± 9.1 ***	55.0 ± 16.4
*At*RA [ng mL^{-1}]	1.77 ± 0.45	1.38 ± 0.42 **	1.47 ± 0.37 §

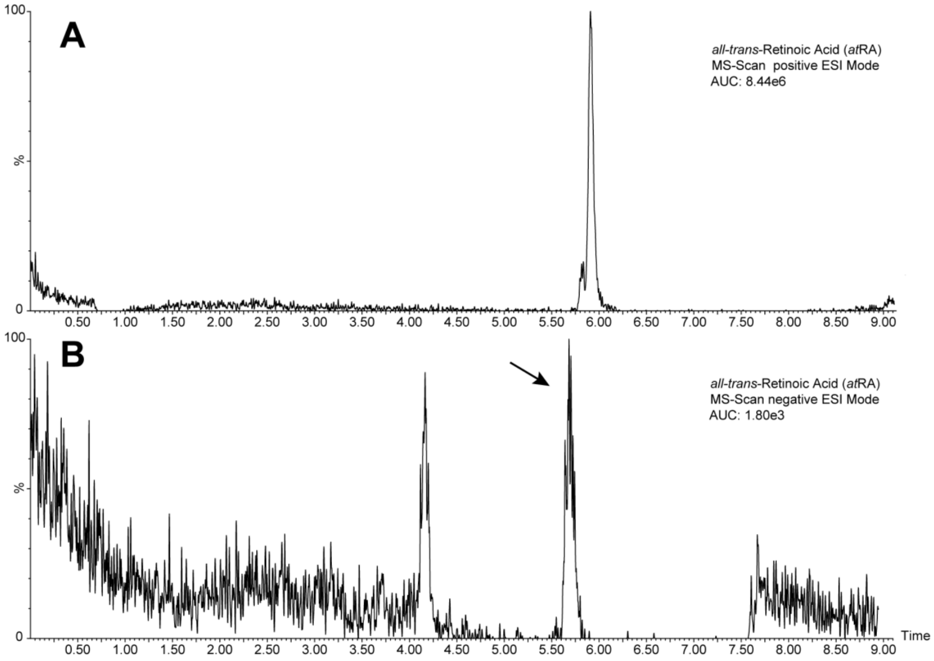

Supplementary Figure 1. Comparison of ionization mode of mass spectrometry for *at*RA (100 pmol direct injection on column). (A) Total ion chromatogram of *at*RA with positive MS-Scan. (B) Total ion chromatogram of *at*RA with negative MS-Scan. The arrow indicates the appropriate peak for *at*RA.
